# The dynamics of behavior in modified dictator games

**DOI:** 10.1371/journal.pone.0176199

**Published:** 2017-04-27

**Authors:** Jeannette Brosig-Koch, Thomas Riechmann, Joachim Weimann

**Affiliations:** 1 Faculty of Economics and Business Administration, University of Duisburg-Essen, Essen, Germany; 2 Faculty of Business Studies and Economics, University of Kaiserslautern, Kaiserslautern, Germany; 3 Faculty of Economics and Management, University of Magdeburg, Magdeburg, Germany; Middlesex University, UNITED KINGDOM

## Abstract

We investigate the dynamics of individual pro-social behavior over time. The dynamics are tested by running the same experiment with the same subjects at several points in time. To exclude learning and reputation building, we employ non-strategic decision tasks and a sequential prisoners-dilemma as a control treatment. In the first wave, pro-social concerns explain a high share of individual decisions. Pro-social decisions decrease over time, however. In the final wave, most decisions can be accounted for by assuming pure selfishness. Stable behavior in the sense that subjects stick to their decisions over time is observed predominantly for purely selfish subjects. We offer two explanation for our results: diminishing experimenter demand effects and moral self-licensing.

## Introduction

The investigation of social or “other-regarding” behavior has been a major topic of experimental economics during the last three decades. In the course of this research, a number of theories integrating some form of other-regarding concerns into the individual preference model have been developed. For example, the models developed by [1] and by [2] are based on the supposition that people are interested not only in their own absolute payoff, but also in their own relative payoff. [3] propose a model of social preferences in which they assume that subjects care about their own payoff, the others’ payoff, and about efficiency. [4] and [5] model a concern for altruism and efficiency by defining utility functions for keeping for oneself and giving to others. Alternative approaches assume some kind of reciprocity caused by the harming or helping intentions of the fellow players (see, e.g., [6], [7], [8], [9], [10]). [11] combine the model of [4] with different forms of reciprocity. What all these models have in common is that they implicitly assume that social behavior is stable over time, i.e. that people make identical decisions in identical repetitions of the decision task. If behavior varies with repetition, these variations are usually attributed to strategic considerations such as reputation building or to some form of learning from information feedback (for an overview, see [12]). But what do the dynamics of social behavior look like if these considerations are excluded? Are there any other influences that cause social behavior to change over time? Although there exists a large body of literature on social behavior, these questions have rarely been addressed before (see the overview below).

In this study, we investigate the dynamics of social behavior in a laboratory environment typically used to obtain evidence on the existence of social behavior. The observation that subjects hand over money to their fellow player in dictator experiments is considered to be one of the most important pieces of proof for the existence of social preferences (e.g., [13]). For this reason, our analyses specifically focus on decisions made in modified versions of the dictator game introduced by [4]. The games are played in a within-subject design using a double-blind procedure. We repeat the experimental sessions with the same dictators twice with a period of four weeks between each repetition. In the following, we label the baseline session as "wave 1", the first repetition as "wave 2", and the second repetition as "wave 3". For each repetition, recipients are newly recruited and dictators are informed accordingly. To check robustness, we also implement sequential prisoner’s dilemma games using the strategy method.

We use the dictator game for at least two reasons. First, these games are simple, i.e. monetary payoff consequences can be inferred directly without any calculation. Second, there is no interdependency of decisions and, therefore, no need for strategic considerations. Accordingly, decisions made in this game can be interpreted as a pure expression of individual (social) preferences. Most importantly, given our experimental design, two prominent explanations for behavioral changes over time, strategic concerns (e.g., reputation building) and learning (e.g., based on feedback about opponents' behavior or about resulting monetary payoffs), can be excluded. Moreover, our use of a double-blind procedure means direct demand effects caused by the experimenter’s observation of subject behavior are not possible in our setting.

If the same subjects play different games within one experiment, it is a natural question whether their behavior is also *consistent* across these games. Although the consistency of behavior is not the main focus of this paper, we also analyze our data in this respect. We use notions of consistency based on the classic selfish approach and on two prominent variants of other-regarding preferences, inequality aversion (see, e.g., [1], and [2]) and altruism (see, e.g., [4]). We transform these models into very general versions which capture the fundamental ideas underlying each approach, but do not make use of particular parameter constellations (see e.g., [14], who estimate the parameters of the [1] model and test the consistency within and across games). This allows us to test whether subjects in our experiments meet the minimal requirements of a theory that assumes rational behavior given that subjects are motivated by altruism or inequality aversion. Because it is not in the center of our interest, we present the consistency analysis in S1 and S2 Files (also see [13], who relate punishment in the ultimatum game to behavior in the dictator game).

We choose to repeat the experimental sessions themselves and not the games within one session because we are particularly interested in the dynamics of social behavior when repetitions can be considered as more disjunctive events (as is often the case in the field). When repeating games within one session, for example, the opportunity costs of participating might decrease over the course of repetitions, while these costs can be assumed to stay rather constant if sessions are repeated under identical conditions. If subjects take opportunity costs (their own and those of their fellow player) into account when making their dictator decision, this may cause behavioral differences between the two modes of repetition. In the following, we relate our design more closely to that of other experimental studies which explicitly or implicitly address the dynamics of social behavior in non-strategic tasks.

### Related literature

Closest to our investigation and most relevant is the work of [15], which tests the impact of the second-to-fourth digit ratio on dictator game giving. In their experiment, 129 undergraduate students of economics played a dictator game twice with a period of seven months in-between. Both sessions were conducted in classrooms. The first session was conducted in the first week of the first academic year. Therefore, the group of students can be characterized as a group of strangers, or at least as people who do not know each other well. The second session was run at the end of the academic year. [15] observed that the amounts of money handed over to recipients in the second session were much smaller than in the first session. There was a small but significant gender effect in that women reduced their giving somewhat less than men ([16] investigate this effect in more detail.). [15] attribute the strong reduction in giving to learning. In the seven months between the two sessions, students might have become familiar with each other as they attended their first courses in economics together, for example. So it is a plausible argument that this learning is the reason for the observed decay in giving to recipients. Our experiment differs from the design used by [15] insofar as we, among other things, randomly recruited subjects from different fields and levels of study via ORSEE, ran the experiment in the laboratory by using a double blind procedure, and newly recruited recipients for each wave (in the experiment by [15], it was randomly determined whether subjects receive money from their dictator decision or whether they receive the gift from another dictator). In doing this, we tried to ensure that the decision situations of the three waves were as far as possible identical. Accordingly, we expect that the kind of learning suggested by [15] is less decisive in our experiment. We will come back to this point later when we discuss our results.

There are also a few experiments in which a dictator game is played more than once within one session. The first study we are aware of that possesses this feature was conducted by [17]. In their experiment, subjects made dictator decisions in a seller-buyer context before and after receiving either “relevant information” (i.e. learning about the allocation made by one other subject) or “irrelevant information” (i.e. learning about the day of birth of one other subject). In their design, all subjects made the two decisions as a dictator (seller), but received their payoff either as a buyer or as a seller in only one of the two games. As such, the dictator games might not be fully considered as repeated games. Nevertheless, the results are relevant and interesting for our experiment. Most importantly, [17] observe that subjects, on average, become more selfish after receiving irrelevant information, but do not when they are informed about another subject’s choice. The authors conclude that “*When individuals are not completely certain about what is the right behavior in a particular context*, *they may regard others' choices in similar situations as sources of useful information*.” (p262). In their experiments on delegation, [18] include a baseline treatment in which subjects played a dictator game 12 times. As in [17], only one of the 12 games was randomly selected for payoff. The reported results suggest that the amounts given to recipients decrease over time. In a second experiment, which was run roughly one year after the first one, the authors implemented a similar baseline treatment with different subjects. In the baseline treatment of the second experiment, there was no such decline of average dictator gifts. However, in the first round of this later baseline treatment, amounts already started at an average level of giving which was almost equal to that observed in the last round of the baseline treatment in the first experiment. The authors do not elaborate on the observed dynamics of dictator game giving. In his meta-study on the dictator game covering 129 papers, [19] finds that, when the game is played repeatedly, dictators offer lower amounts and equal splits become less likely. [20] conduct an experiment in which 16 dictator games were played within one session. The 16 games differed with respect to the information subjects (dictators/recipients) were provided about their opponents. As in the previous experiments, only one randomly selected game was paid off. [20] observe that in this setting neither the framing of the games nor the number of games already played had an impact on dictator decisions. But they detect systematic moral self-licensing and moral cleansing patterns, an observation which we will refer to later in our discussion. In the experiment by [21], subjects played a dictator game 12 times (after they were confronted with another task manipulating their self-control). Incentives were such that a subject received his or her proposed dictator share for 6 randomly chosen games and acted as a recipient of dictator gifts for the other 6 games. [21] observe that dictator gifts decrease over time and hypothesize that subjects might become more familiar with the decision situation over time, which might eliminate potential demand effects and possibly reduce image concerns. We will come back to this hypothesis in our discussion of results.

[22] also investigate a kind of repeated decision. Although they use an ultimatum game, not a dictator game, their results may be of interest. In their experiment, the second mover had a second chance to think about her decision. Having decided whether she wanted to accept the offer of the proposer or not, she had to fill out a questionnaire. On finishing, she was asked if she wanted to change her decision. It turned out that the rejection rate was significantly smaller in the treatment which offered the second chance than in the treatment in which this was not the case. Once again, this can be interpreted as some kind of learning. But we would prefer a different explanation. [23] investigate a game in which a second player can choose to punish the first mover at his own cost. They find that the punishment rate was much lower in the cold version of the experiment, in which the strategy method was used, than in the hot version, in which second movers had to react directly to an unfriendly move of the first player. This indicates that punishing is an emotional act carried out the moment one is confronted with an annoying move by another person. The opportunity to think it over has a cooling effect and this might explain the lower rate of punishment in the second chance treatment of [22].

In a recent study, [24] apply our multi-wave design to test the temporal stability of behavior in a one-shot public-good game. In their strategic decision environment, they find that cooperation decisions appear to be quite stable at the aggregate level. [25] support this finding in their investigation of contributions made to public goods in rural Vietnam. Note that players are involved in a strategic interaction in a public good game, which is not the case in a dictator game. This makes the two games hard to compare.

Our paper proceeds as follows. Section 2 includes a detailed description of the games we use in our study and section 3 describes how the games were implemented in the experimental design. The results are presented in section 4. In section 5, we will discuss two possible explanations for our results and embed this discussion in the relevant literature. The first explanation is concerned with the relevance of experimenter demand effects in our setting (see, e.g., [21]). The second refers to moral licensing effects (see, e.g., [20]). Section 5 also discusses the question of how our findings relate to the ongoing debate about the external validity of experimental results.

## The games

Our experimental design is inspired by the studies conducted by [26], who use the modified dictator games introduced by [4]. As a control, we use sequential prisoner’s dilemma games first employed by [27]. The games are described in detail below.

### Modified dictator games

In order to investigate the dynamics of pro-social behavior, we primarily use modified dictator games because these games offer two advantages. First, they do not leave any room for strategic considerations and can be used to directly observe dictators' pro-social concerns. Second, they can be presented in different variants. In total, we employ eight different dictator games. This allows us to check whether the behavioral dynamics observed in the course of the three waves are valid for all eight variations. In each game, the dictator has to choose from eleven different distributions of payoffs to himself, *π*_*A*_, and to the recipient, *π*_*B*_. There are two different types of dictator games used in the experiment: ‘take’ games and ‘give’ games. Note that [28] and [29] use a somewhat related modification of dictator games. In their games, the subjects’ action set is extended in a way that allows them to either give money to or take money from the recipient. They find that this variation significantly decreases the number of subjects giving a positive amount.

#### Take games

In each of the four take games, starting from the initial endowment (*π*_*A*_^*E*^, *π*_*B*_^*E*^) = (500, 500), player A (the dictator) can reduce player B’s (the recipient) payoff by d*π*_*B*_ in order to increase his own payoff by d*π*_*A*_ at a constant relative price of *p*_*A*_ = |d*π*_*B*_ / d*π*_*A*_|, such that *π*_*A*_ = 500 + (1 / *p*_*A*_) (500 − *π*_*B*_). This budget constraint can be re-formulated as *π*_*B*_ = 500 + *p*_*A*_ (500 − *π*_*A*_). Accordingly, the ‘budget line’ has a slope of–*p*_*A*_. The four games only differ with respect to this slope: in the first game, T1, we have *p*_*A*_ = *p*_*A*_^T1^ = 1/2; in the remaining games, the values are *p*_*A*_^T2^ = 2/3, *p*_*A*_^T3^ = 1, and *p*_*A*_^T4^ = 2, respectively. Except for the equal payoff distribution, all possible options in the take games are chosen in such a way that player A is assured of a higher payoff than player B, i.e. *π*_*A*_ > *π*_*B*_. The experimental set-up for the four games is illustrated in Table 1.

**Table 1:** Payoffs in the four take games.

Game	*π*	1	2	3	4	5	6	7	8	9	10	11
T1	*π*_*A*_ *π*_*B*_	500, 500	600, 450	700, 400	800, 350	900, 300	1000, 250	1100, 200	1200, 150	1300, 100	1400, 50	1500, 0
T2	*π*_*A*_ *π*_*B*_	500, 500	575, 450	650, 400	725, 350	800, 300	875, 250	950, 200	1025, 150	1100, 100	1175, 50	1250, 0
T3	*π*_*A*_ *π*_*B*_	500, 500	550, 450	600, 400	650, 350	700, 300	750, 250	800, 200	850, 150	900, 100	950, 50	1000, 0
T4	*π*_*A*_ *π*_*B*_	500, 500	525, 450	550, 400	575, 350	600, 300	625, 250	650, 200	675, 150	700, 100	725, 50	750, 0

#### Give games

In each of the four give games, starting from the initial endowment (*π*_*A*_^*E*^, *π*_*B*_^*E*^) = (500, 500), player A (the dictator) can increase player B’s (the recipient) payoff by d*π*_*B*_ at a personal cost of d*π*_*A*_ at a constant relative price of *p*_*A*_ = |d*π*_*B*_ / d*π*_*A*_|, such that *π*_*A*_ = 500 + (1 / *p*_*A*_)(500 − *π*_*B*_). Again, this budget constraint can be re-formulated as *π*_*B*_ = 500 + *p*_*A*_ (500 − *π*_*A*_). Accordingly, the ‘budget line’ has a slope of d*π*_*B*_ / d*π*_*A*_ = –*p*_*A*_. The four games only differ with respect to this slope: in the first game, G1, we have *p*_*A*_^G1^ = 2; in the remaining games, the values are *p*_*A*_^G2^ = 3/2, *p*_*A*_^G3^ = 1, and *p*_*A*_^G4^ = 1/2, respectively. Choices in the give games (except for the equal payoff distribution) grant a higher payoff to player B than to player A, i.e. *π*_*A*_ < *π*_*B*_. The experimental set-up is illustrated in Table 2.

**Table 2:** Payoffs in the four give games.

Game	*π*	1	2	3	4	5	6	7	8	9	10	11
G1	*π*_*A*_ *π*_*B*_	500, 500	450, 600	400, 700	350, 800	300, 900	250, 1000	200, 1100	150, 1200	100, 1300	50, 1400	0, 1500
G2	*π*_*A*_ *π*_*B*_	500, 500	450, 575	400, 650	350, 725	300, 800	250, 875	200, 950	150, 1025	100, 1100	50, 1175	0, 1250
G3	*π*_*A*_ *π*_*B*_	500, 500	450, 550	400, 600	350, 650	300, 700	250, 750	200, 800	150, 850	100, 900	50, 950	0, 1000
G4	*π*_*A*_ *π*_*B*_	500, 500	450, 525	400, 550	350, 575	300, 600	250, 625	200, 650	150, 675	100, 700	50, 725	0, 750

#### Sequential prisoner’s dilemma games

**Fig 1:**
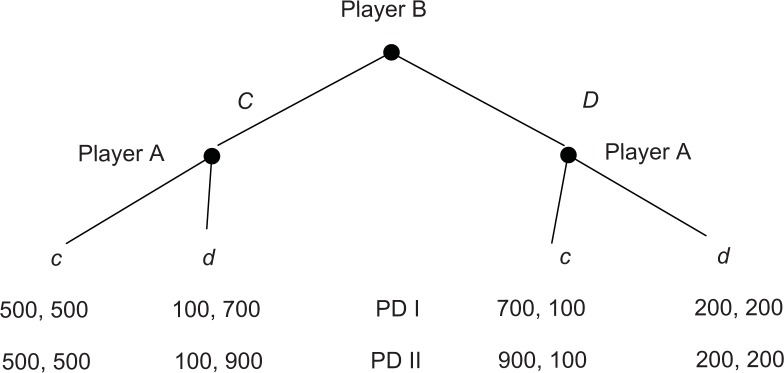
Payoffs in the two prisoner’s dilemma games [*π*_*B*_, *π*_*A*_].

We use dictator games because they exclude strategic considerations. But of course it would be interesting to know whether a particular dynamic of pro-social behavior can also be observed in an environment with strategic aspects. Therefore, we use a simple sequential prisoner’s-dilemma experiment as a control treatment. The payoffs in our two sequential prisoner’s dilemma (PD) games are given in Fig 1. In both games, the decisions of player A (the second mover) are elicited using the strategy method, i.e. player A has to respond to each of the two actions feasible for player B (the first mover).

We make use of the strategy method because this allows us to exclude any feedback about the opponent’s behavior and, thus, eliminates all direct strategic considerations. As, for example, [23] have shown, applying this method might possibly influence behavior (for an overview, see [30]). Note, however, that even if this influence exists in our experiment, it is the same for all of our games since all of them are played in a “cold” mode.

**Fig 2:**
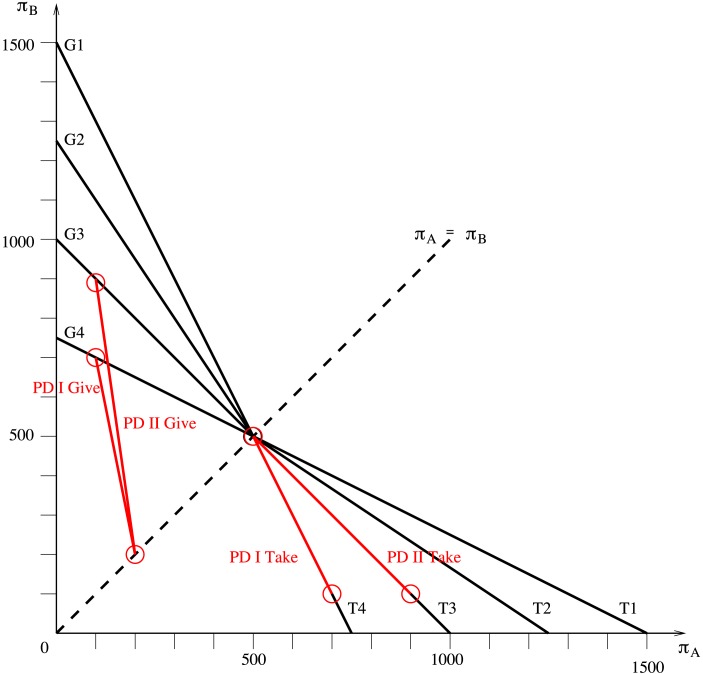
Budget lines in modified dictator and sequential prisoner’s dilemma games.

Note that the PD-subgames played by A can be interpreted as mini take and give games, where the take games are the ones following player B’s *C*-move and the give games are the ones following player B’s *D*-move. The mini take game in PD I leaves player A the choice between (*π*_*A*_^*E*^, *π*_*B*_^*E*^) = (500, 500) and (*π*_*A*_, *π*_*B*_) = (700, 100). This creates a relative price of *p*_*A*_ = 2, such that the slope of the respective budget line is d*π*_*B*_ / d*π*_*A*_ = -2 (similar to game T_4_). In the mini take game of PD II, the slope of the respective budget line is d*π*_*B*_ / d*π*_*A*_ = -1 (similar to game T_3_). The mini give game in PD I gives player A the opportunity to choose between (*π*_*A*_^*E*^, *π*_*B*_^*E*^) = (200, 200) and (*π*_*A*_, *π*_*B*_) = (100, 700). This creates a relative price of *p*_*A*_ = 5, such that the slope of the respective budget line is d*π*_*B*_ / d*π*_*A*_ = -5. In the mini give game of PD II, the slope of the budget line is d*π*_*B*_ / d*π*_*A*_ = -7. The budget lines in the modified dictator games and sequential prisoner's dilemma games are illustrated in Fig 2.

## Experimental design

The ten games (four take games, four give games, and two prisoner’s dilemma games) were played in two sessions within one week. In session 1, all of our 40 dictator subjects participated in the four take games and in prisoner’s dilemma I. In session 2, the same 40 subjects participated in the four give games and in prisoner’s dilemma II. The sequence of play is illustrated in Table 3. We opted to implement an identical order of tasks for all 40 players, as this allows us to study the dynamics of their behavior in a highly controlled manner, i.e. based on an identical order of play. To get a similarly high level of control in a random order design, we would have to recruit a much higher number of subjects (which would have been more difficult since subjects had to sign up for an unknown number of future experiments). The two sessions were run with four groups of ten players A (dictators) in a within-subject design. In addition, we also recruited four groups of ten players B for each session.

**Table 3:** Sequence of play.

	1st game	2nd game	3rd game	4th game	5th game
Session 1	T2	T4	PD I	T1	T3
Session 2	G3	G1	PD II	G4	G2

The two sessions (each consisting of the five games included in Table 3) were repeated twice with the subjects in the role of player A, once after four weeks (wave 2) and once after another four weeks (wave 3). Conducting the 2 x 3 sessions using a within-subject design for players A allows us to investigate the temporal dynamics of their behavior. When signing up for the first two sessions, players A were told that they would possibly have to take part in more than one experiment, but were left ignorant about the number of experiments they would have to participate in. Players B were newly recruited for each session and each wave and players A were informed accordingly. This design feature should eliminate strategic considerations that could influence the dynamics of behavior.

At the beginning of each session in waves 1 and 2, the subjects were told that they had to make decisions, but were left ignorant about the structure and number of games to be played (instructions are included in S4 File). Wave 3 differed from the previous two waves in that the subjects were informed about the five games immediately at the beginning of each session. Changing the informational design in this direction, we mimic the experience-enhancing effect of repeating the experiment. Potentially this change favors non-selfish behavior. Given a subject plans to ‘give’ some money to one of his opponents, he can choose the one single game within the session that suits him most to do this (maybe because giving to others is particularly ‘cheap’ in that game, maybe because the game allows the subjects to give away a certain amount of money, or for other reasons). Calculating the total amount of money players A allocate to themselves or the total amount of money players A allocate to their opponents for all three waves separately, we do not observe such an increase in other-regarding behavior, however (see Figs A and B in S3 File). In all sessions, the subjects had to submit their choices one after the other.

We employed a perfect random matching design in all sessions, i.e. players A were matched in each game with different players B, and the subjects were informed accordingly. In addition, the subjects were told that they would receive no feedback about their partner’s and others’ decisions during the experiment. The purpose of this design was to prevent players A from “learning” about their opponents’ behavior or from the behavior of the other dictators (see [17], who study the influence of information about others’ choices in a dictator game). Nevertheless, players A could possibly compute their opponents’ (past) behavior in PD-games from their total profits and could condition their behavior in the next games, played with new opponents, on their former opponents’ choices. Such behavior is not observed in our data set, however. At the end of each session, subjects were paid off the total profit they had made in the five games at an exchange rate of 150 lab-cents = 100 eurocents (whenever ‘Cent’ is used in the following, ‘lab-cent’ is meant). The payment was conducted anonymously employing a double-blind procedure.

The computerized experiment was run with a total of 270 participants who were students of different subjects at the University of Magdeburg, Germany. To recruit the subjects, we used the web-based Online Recruitment System ORSEE, which is specifically designed for organizing economic experiments ([31]). Written informed consent was obtained by all subjects. In wave 1, there were 40 (40) players A (B) in both sessions. Due to no-shows, in wave 2 there were 39 (39) players A (B) in both sessions, and in wave 3 there were 37 (37) players A (B) in session 1 and 35 (35) players A (B) in session 2. The following analyses are based on the 35 players A who took part in all sessions in all three waves. The experiment was computerized using [32]’s z-Tree software tool. As the experiment was run using a double-blind procedure, we did not collect any identifying information from the participants. Average payoffs per session were €15.03, with a minimum of €0.67 and a maximum of €34.67. There was no show-up fee. No session lasted longer than 30 minutes.

## Results

In the following we present the results of our experiment. Our analyses are based on the 35 subjects who participated in all three waves. All the individual data are included in S5 File. Table A in S5 File includes the individual choices of *τ* (the amount taken from player B) in the take games, Table B in S5 File includes the individual choices of γ (the amount given to player B) in the give games, and Table C in S5 File includes the individual choices made in the PD games.

### Behavior in the dictator games

**Fig 3:**
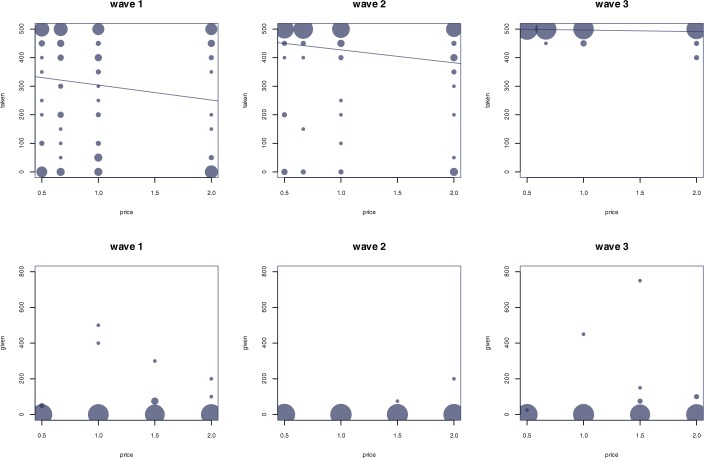
Amounts taken from players B in the take games (the upper 3 graphs) and amounts given to players B in the give games (the lower 3 graphs).

Fig 3 illustrates the behavior in the four take games (upper three graphs) and in the four give games (lower three graphs) over three waves. The size of the dots is proportional to the number of observations. The thin lines in Fig 3 display the result of a fixed effects OLS regression of the amounts taken within each wave with the price *p* of player A’s payoff as independent variable.

The size of the dots indicates the number of observations. The thin line in the upper three figures represents the results of a fixed effect OLS regression with *τ* as dependent variable and *p* as independent variable.

#### Take games

In the take games, we observe that dictators, on average, do not take the maximum amount in wave 1, but leave a considerable amount of money for recipients. The average amount taken, if anything, significantly decreases with a higher relative price for the payoff of players A (*p* ≤ 0.021 for T2 vs. T4 and T2 vs. T3, *p* = 0.098 for T1 vs. T4, *p* ≥ 0.129 for the other three game comparisons; if not reported otherwise, two-tailed Wilcoxon signed-rank test are used). Apparently, dictators tend to take more if the loss of the recipient per Cent taken is smaller. A similar relationship between the amounts taken and the price *p* can be observed in wave 2 (*p* ≤ 0.028 for T1 vs. T4, T2 vs. T4, and T3 vs. T4, *p* = 0.078 for T2 vs. T3, *p* ≥ 0.375 for the other two game comparisons), but not in wave 3 (p > 0.100). The observed differences between the take games are in line with general concepts of altruism or inequality aversion (see S1 File for more details).

Over the three waves, we observe a sharp increase in the average amount taken from player B, though. While the average amount taken per game is 301.8 Cents in wave 1 (remember that the maximum amount that could be taken is 500 Cents per game), this amount significantly increases to 425.4 in wave 2, and significantly increases further to 496.4 in wave 3 (differences are labeled as significant if *p* < 0.050 and are labeled as weakly significant if 0.050 ≤ *p* < 0.100). We also run a Jonckheere-Terpstra test (see [33]) to check whether the increase in the amounts taken is significant. The null hypothesis of this test is that the distribution of fractions taken does not differ among waves. The alternative hypothesis is that there is an ordered difference between waves (factions increase). We have to reject the null hypothesis and this result is highly significant (*p* < 10^−15^). In the last wave, there is no subject who takes less than, on average, 450 Cents per game. Out of the 35 subjects, 28 never decrease the amount taken over the three waves (i.e. they either stick to their previous choice or increase the amount taken from one wave to the other). Only seven subjects decrease the amount taken at least once over the three waves (the ten decreases made by these subjects account for only 3.6 percent of the 35subjects x 4games x 2repetitions = 280 potential changes that could be made over time).

To separate the decision to behave strictly selfishly or not from the choice of a specific amount taken, we apply a Cragg hurdle model. The likelihood function for the overall hurdle model is constructed as the product of two likelihoods, the likelihood that the dictator takes the maximum amount or not (probit) and the conditional likelihood that the dictator takes something from player B (see, e.g., [34] for more details). To treat observations as independent across subjects, but not within, we use a clustering specification. Explanatory variables include binary dummy variables for games T2, T3, and T4 and for wave 2 and wave 3. Table 4 presents our results. The second column refers to the decision to take the maximum amount or not. The third column refers to the amount taken conditional on that not everything is taken.

**Table 4:** Hurdle model on the amount taken from player B.

	Logit Participate	Truncated linear regression Tau
Wave 2	0.865***	117.5
	(0.187)	(86.02)
Wave 3	1.947***	958.2***
	(0.284)	(203.0)
T2	-0.0463	97.89**
	(0.103)	(47.29)
T3	-0.444***	77.15**
	(0.119)	(38.86)
T4	-0.636***	-1.032
	(0.167)	(54.69)
Constant	500.0***	181.4**
	(0.196)	(71.87)
Observations	420	135
Cluster per subjects	yes	yes

Notes: Hurdle model on amount taken from Player B. Standard errors in parentheses, clustered at subjects level. Wald test has Chi2(5) = 25.68 (p-value = 0.001).

***Significant at the 1 percent level

**Significant at the 5 percent level

* Significant at the 10 percent level

Table 4 reveals that repeating a specific game has a significantly positive effect on the likelihood to take everything in this game. In addition, dictators are significantly more likely to take higher amounts when the game is played the third time with this conditional on their choosing not to take the maximum amount. Increasing the relative price for player A’s payoff in T2, T3, and T4 compared to T1 also affects both the decision to take everything or not and the amount taken in the case that not everything is taken. In particular, increasing the price from T1 to T3 or T4, respectively, significantly decreases the likelihood of taking the maximum amount from player B. But, conditional on not everything being taken, a price increase from T1 to T2 or to T3, respectively, seems to make taking *higher* amounts more likely. The latter effect does not show up when focusing on unconditional taking.

The results from applying an ordered probit regression or an ordered logit regression on dictators’ choices using the same explanatory variables as for the hurdle model are included in Tables A and B in S6 File. The results for both models reveal that playing the game a second or a third time has a significantly positive effect on the amount taken, while increasing the relative price for player A’s payoff from T1 to T3 or to T4 has a significantly negative effect. Note that using other specifications in these models yields very similar results.

#### Give games

In the give games, we observe that, on average, dictators already give almost nothing to recipients in wave 1, i.e. players B receive, on average, 8.57 Cents out of the maximum possible amount of 1000, and this behavior does not significantly change over time (wave 2: 5.71 Cents, wave 3: 5.71 Cents). More specifically, while 29 out of the 35 subjects either choose to give nothing in all games and in all waves (23) or reduce their gifts to zero after wave 1 (6), the rest switches from giving less to giving more in the course of the three waves at least once. Moreover, the fact that giving creates an efficiency gain in G1 and G2 does not significantly influence decisions. In particular, in none of the three waves are there significant differences between the four give games. Applying regressions similar to those used for the take games reveals only weak effects, if at all. In particular, playing the game a second time tends to negatively affect the decision to give, while increasing the relative price for player A’s payoff from G1 to G2 tends to positively affect this decision, if at all (see Tables C, D, and E in S6 File for more details).

Recall that giving has two effects in the give games: it reduces the payoff the dictator receives and, at the same time, it increases the inequality between the two players. Accordingly, giving nothing in these games is in line with the assumption that the subjects are inequality averse.

### Behavior in the prisoner’s dilemma games

**Fig 4:**
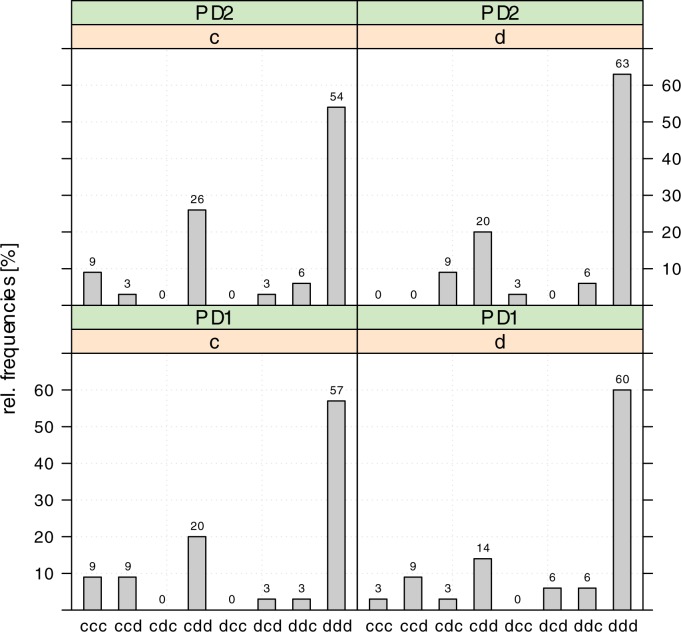
Relative frequencies of individual behavioral patterns over the three waves.

We employed the sequential prisoner’s-dilemma games to check whether the dynamics observed in modified dictator games differ in a more strategic setting. In the two PD games, players A act as second movers and are, thus, once again in the role of a dictator. Fig 4 illustrates the dynamics of individual behavior for each of the two subgames of PD I and PD II. The three letter combinations on the horizontal axis represent all possible combinations of moves that can be observed for each player A in each subgame over the three waves. Accordingly, each column illustrates the relative frequency of subjects revealing the specific dynamics of moves in the subgame over the three waves.

Fig 4 reveals that individual behavioral patterns do not differ much between the four subgames. Applying a 2-sample test for equality of proportions with continuity correction, we do not find significant differences between the four subgames. While this independency of decisions observed in our strategy method version of the sequential prisoner's dilemma game is at odds with the observations made by, e.g., [27], it is still in line with inequality aversion and altruism (see S1 File). In all subgames, the majority of the subjects (54 to 63 percent) choose *d* in each of the three waves. The second most frequent pattern we observe in each subgame is that the subjects start by choosing *c* in wave 1 and end by choosing *d* in wave 3 (20 to 29 percent). It is observed rather rarely in each of the four subgames that the subjects stick to choosing *c* in each wave (0 to 9 percent) or start by choosing *d* in wave 1 and end by choosing *c* in wave 3 (3 to 9 percent). Overall, in the PD games we find similar dynamics to those in the modified dictator games. While over 77 percent of the subjects (27 out of the 35) choose to either behave strictly selfishly in all games and in all waves or reduce their social engagement after wave 1, only a small fraction (8 out of 35) switch from a *d* to a *c* move in the course of the three waves at least once. There is no subject choosing *c* in all the games and in all three waves.

A logit regression that controls for the PD game, the subgame, and the wave supports our previous results. While neither the game nor the subgame have a significant influence on the likelihood to choose *d*, there is a significantly positive effect of the waves on this likelihood (see Table F in S6 File). Applying a probit regression reveals very similar results (see Table G in S6 File).

## Discussion

In our study, only selfish subjects do not deviate from their initial decision over the course of the three waves. The vast majority of those subjects starting with pro-social decisions in wave 1 change these decisions into more selfish ones and offer less to recipients in subsequent waves.

The observation that the tendency towards more selfish behavior is pronounced in all variants of the dictator game and also in the two PD games provides a strong argument that this tendency is a systematic behavioral pattern. Of course, we do not know what happens to our subjects between their visits to the laboratory (which is a valid argument that applies to all existing studies including repeated sessions). As the subjects are randomly recruited from different fields and levels of study and players B are newly recruited for each wave, learning in the sense that players A and B get acquainted with each other over the course of the experiment (as might have been the case in [15]) can hardly explain our observations. Moreover, in the controlled environment of the laboratory, the subjects made their decisions anonymously and without feedback, with the result that they could not identify each other’s choice. Nevertheless, even if players A had talked about their choices afterwards, the findings by [17] suggest that such observations of others’ behavior would have reduced the tendency towards more selfishness. It may, however, be that the different experiences of the subjects between the waves could have affected their behavior in the next wave. But it is hard to argue that all these different influences should lead to more selfishness. Instead, if these different experiences were really relevant for the subjects’ decisions, we should have observed much more noise in our data on the dynamics of decisions. But this was not the case. So what causes the systematic behavioral change to more selfishness over the three waves?

As already mentioned, our experiment leaves only little room for learning about other subjects. But there are other types of *learning* that can affect behavior. First, subjects can learn how to play the game by learning the rules of the game and by learning how to calculate their best answer. This is the kind of learning without feedback information that [35] refers to when explaining his results. [35] uses a guessing game in which *k*-level reasoning is needed to solve the game. It is highly plausible that a player recognizes his or her own strategy as soon as it is chosen in round *t* and, thus, realizes even without feedback that others in round *t+1* are very likely to behave the same way as he did in round *t*. Accordingly, in round *t*+1 this player optimizes against the strategy he applied in round *t*. But in our experiment we focus on dictator games in which there is no strategic interaction and in which the subject’s own payoff does not need to be calculated. So these games appear to be too simple to leave any room for this type of learning. Second, it might be that subjects learn about the experimental situation over the course of the three waves. For example, they might learn about how the experimenter handles the situation and whether there is any specific “demand” regarding the behavior revealed by subjects (as argued, e.g., by [21]). They might also learn whether there are any kinds of rewards or sanctions that result during or after the session and which follow a certain kind of behavior. Third, subjects might learn about themselves, i.e. about how they feel when they make a particular decision (as argued by, e.g., [20]). As the latter two types of learning might also be relevant in our experiment, we will provide two possible explanations for our observations which are based on these two types.

### Explanation 1: A diminishing experimenter demand effect

Following [36], the most important reason for experimenter demand effects (in the following abbreviated as EDE) is the fact that subjects are uncertain about the decision environment when they enter a laboratory. They do not usually know either the purpose of the experiment or what the “right” or adequate behavior is in this unfamiliar and artificial situation. Because of this fundamental uncertainty, everything implicitly or explicitly communicated by the experimenter will be used as a signal from which subjects try to infer what the experiment is about and how they should behave. These signals include the framing of the experiment, the experimental design, the instructions, the social distance to the experimenter and to the other subjects, and the way the experimenter interacts with the subjects. The whole setup of a standard dictator game experiment might signal to subjects that *this is an experiment in which it is tested how altruistic you are*. That is, from the subjects’ perspective the experimenter might have designed this experimental setting exactly for this purpose. Our conjecture is that, if subjects interpret the experimental situation in this way, most of the giving we observe in wave 1 of our experiment is a reaction to this strong EDE. Some arguments favoring this hypothesis can be derived from the discussion about framing effects in dictator experiments.

Some researchers argue that the dictator experiment is extremely sensitive and prone to framing effects ([36], [37]). For example, it makes a huge difference whether dictators have to work for their initial endowment or not ([38]), whether they can only give money to the recipient or whether they are also allowed to take money from his endowment ([28], [29]), and how the social distance is designed (e.g., [39], [40]). [41], for example, varied the frame by adding the sentence „*RECUERDA el esta en tus manos”*(Remember he is in your hands.) to the instructions. This significantly increased the gifts handed over to the subjects and this effect was even stronger when the experiment was conducted by a professor instead of a research assistant. On the other hand, [42] run a large-scale experiment and do *not* find framing effects in their dictator games. These seemingly contradicting findings can be explained by assuming that different frames go along with different EDEs. For example, if the task is framed as a “give or take game” as in [28] and [29], this framing carries a much different signal than the original dictator game which is framed as a “give game” only. In the former case, subjects might infer this as permission (provided by the experimenter) to consider taking as an eligible option. The frame used by [41] also carries a strong EDE as subjects can infer the signal as “Let’s see if you are willing to exploit someone who is in your hands”. The fact that this frame has a stronger impact if it comes from a professor makes it very likely that an EDE is the driving force for the observed results (see [36] for a similar interpretation of the observations made by [41]).

If giving in dictator experiments is (at least partly) motivated by EDEs and if these EDEs are rooted in subjects’ uncertainty about the decision environment, then these EDEs should diminish when subjects become more familiar with, i.e. when they learn about, the decision environment. There is much room for this kind of learning in our repeated session experiment as the subjects experience that, in fact, decisions are made anonymously and they experience that, in fact, nobody reacts to their decisions either during or after the session. In particular, the subjects learn that there are neither rewards nor sanctions that result from their behavior. If the subjects only give because they feel obliged by EDEs to do so, this giving should decrease over the course of the three waves. In this case “*Pro-social behavior might be a short-lived phenomenon*….” ([21], p10).

### Explanation 2: Moral licensing

There is evidence from psychological research indicating that people feel licensed to refrain from other-regarding behavior if they have behaved in a ‘moral’ manner before. For example, [43] find that subjects who make statements demonstrating a lack of bias against women or minorities are more likely to later behave in a way that conforms to negative stereotypes about the respective group. Other studies on this so-called ‘moral-licensing’ effect include [44], [45], and [46]. [47] provide a recent survey of the moral licensing literature. There is also recent experimental literature referring to moral licensing effects (e.g., [48], [49], or [50]). Most relevant is the experiment by [20] in which the subjects played 16 dictator games within one session with each game framed in a different manner. While [20] neither detected framing effects nor observed a unique change in behavior over the course of play, they report a moral licensing and a moral cleansing effect: after giving money to the recipient, the subjects reduced their offer in the next game (which is in line with moral licensing) and after being selfish, the subjects increased their offers in the next game (which is in line with moral cleansing). Both effects are in line with learning about oneself, i.e. about the feeling that is associated with making a specific decision, which might be offset by making another decision in the subsequent game. Moral licensing seems to be a promising candidate to explain the tendency towards more selfish decisions in our experiment as well. Having already given (perhaps because of strong EDEs), subjects allow themselves to be more selfish in the next wave. Over the course of the three waves, at least, we do not observe a systematic behavioral pattern that would be in line with moral cleansing.

Our conjecture is that the effects described in explanations 1 and 2 are both at work in our experiment. On the basis of our experiment, however, it is not possible to assess their relative strength.

Finally, our results might be also considered from a methodological point of view. Starting with [51] Levitt and List (2007), there is a lively debate on the external validity of experimental research in the laboratory (e.g., [52], [53], [54], [55]) as well as a rapidly growing body of experimental literature directly focusing on basic methodological questions of laboratory research (e.g., [56] re-investigate the effect of the “experimenter-subject” interaction under laboratory conditions; [57], [58], and, more recently, [59] test whether social preferences disappear under high stakes; [60] ask whether student subjects reveal significantly higher degrees of social preferences than non-student subjects; [61] show that there are no systematic differences between student subjects and adults). The experimental results reported in our paper reveal that the repetition of experimental sessions changes behavior to more selfishness. If this change of behavior is (at least to some degree) due to EDEs, the share of pro-social behavior we observe with “inexperienced” subjects in the laboratory might be much higher than what should be expected in reality. In fact, in the field we do not observe that people make unconditional monetary gifts to anonymous strangers, although this is what we usually observe in one-shot dictator experiments. Our experiment suggests the necessity to be much more careful when drawing conclusions regarding the extent to which pro-social behavior is relevant in non-strategic decision tasks outside the laboratory. One way to increase the external validity of dictator-game-like laboratory experiments is to implement repeated sessions or to use subjects who are more familiar with this type of decision making in the laboratory.

## Supporting information

S1 File The analysis of the consistency of behavior. (PDF)

S2 File Behavioral dynamics and consistency. (PDF)

S3 File Figures on the total payoff resulting from the decisions made by players A. (PDF)

S4 File Instructions. (PDF)

S5 File Data. (PDF)

S6 File Regression results. (PDF)
