# Supplementary material for: The dynamics of behavior in modified dictator games
Source: PLoS One. 2017 Apr 27;12(4):e0176199. doi: 10.1371/journal.pone.0176199 (PMC5407812; doi:10.1371/journal.pone.0176199)
Supplement: S1 File — (PDF) [file pone.0176199.s001.pdf]

S1. The analysis of the consistency of behavior

Our concepts of consistency differentiate between three preference types: 'S-consistency' is based on **s**elfish preferences, 'A-consistency' is based on **a**ltruistic preferences, and 'I-consistency' is based on **i**nequality averse preferences. These three concepts of consistency are, by definition, not mutually exclusive, though. There are modes of behavior that can be consistently explained by more than just one of these concepts. All the concepts of consistency refer to decisions made at a particular point in time.

S1.1 Consistency according to selfish preferences

A player A with selfish preferences cares solely about his own payoff π_A. Assuming that player A derives positive utility from π_A, i.e.

$$u_t^S(\pi_A) \quad \text{with} \quad \frac{\partial u_t^S(\pi_A)}{\partial \pi_A} > 0,$$

then a selfish individual will always maximize his own payoff. The implications for the definition of 'S-consistency' in our games are summarized in Table A. An S-consistent player A will always take the maximum possible amount from player B in the take games (i.e. will always choose option 11), will always transfer the minimum possible amount to player B in the give games (i.e. will always choose option 1), and will always choose *d* in PD games.

By definition, all types of other-regarding preferences include selfish preferences as a special case.

S1.2 Consistency according to inequality aversion preferences

Our second concept of consistency takes into account notions of inequality aversion. Preferences of this type have been introduced by [1] and [2]; [3] also consider inequality aversion, but additionally include reciprocity concerns in their preference model. Note that, in our games, considering reciprocity along the lines of [3] does not change the predictions for individual behavior. In particular, the specific concepts by [3] and by [1] would lead to the same results in our games:

For a two-person game with players A and B, [1] specify individual A's preferences concerning his own payoff π_A and his opponent's payoff π_B:

$$U_A = u(\pi_A, \pi_B) = \begin{cases} \pi_A - \beta(\pi_A - \pi_B) & \text{for} \quad \pi_A \geq \pi_B \\ \pi_A - \alpha(\pi_B - \pi_A) & \text{for} \quad \pi_A < \pi_B \end{cases}.$$

The corresponding specification by [3], p.822 reads

$$U_A = u(\pi_A, \pi_B) = \begin{cases} (\rho + \theta q)\pi_B + (1 - \rho - \theta q)\pi_A & \text{for} \quad \pi_A \geq \pi_B \\ (\sigma + \theta q)\pi_B + (1 - \sigma - \theta q)\pi_A & \text{for} \quad \pi_A < \pi_B \end{cases}$$

where q is an indicator variable signaling the presence of reciprocity. In the case that the opponent, player A, “misbehaves” ([3], p. 822), q becomes $q = -1$. As we designed our experiments in a way that avoids reciprocity, we can, for our paper, set $q \equiv 0$, which leads to the simple specification

$$U_A = u(\pi_A, \pi_B) = \begin{cases} \rho\pi_B + (1 - \rho)\pi_A & \text{for} \quad \pi_A \geq \pi_B \\ \sigma\pi_B + (1 - \sigma)\pi_A & \text{for} \quad \pi_A < \pi_B \end{cases}.$$

This can be re-written as

$$U_A = u(\pi_A, \pi_B) = \begin{cases} \pi_A - \rho(\pi_A - \pi_B) & \text{for} \quad \pi_A \geq \pi_B \\ \pi_A + \sigma(\pi_B - \pi_A) & \text{for} \quad \pi_A < \pi_B \end{cases}.$$

This form shows that, in our games, the specification by [1] and the one by [3] are equivalent for $\rho = \beta$ and $\sigma = -\alpha$.

Since sequential prisoner’s dilemma games represent strategic interactions, reciprocity might play a role. According to [3], reciprocity only matters, however, if player B ‘misbehaves’, i.e. if player B chooses *D*. Following the *D*–move, player A can achieve both a higher π_A and a lower difference between π_A and π_B by choosing *d*. That is, utility increases even more when considering reciprocity along the lines of [3].

In the following, we present a more general notion of inequality aversion which is inspired by the three papers above. With preferences including inequality aversion, a player gains utility from his own payoff and loses utility from a difference between his own and the other’s payoff. The utility function can be written as follows

$$u_t^I(\pi_A, \Delta\pi) \quad \text{with} \quad \Delta\pi = \begin{cases} \pi_A - \pi_B & \text{for} \quad \pi_A - \pi_B \geq 0 \\ \pi_B - \pi_A & \text{for} \quad \pi_B - \pi_A > 0 \end{cases} \quad \text{and with} \quad \frac{\partial u_t^I(\pi_A, \Delta\pi)}{\partial \Delta\pi} \leq 0.$$

The implications for the definition of ‘I-consistency’ in our games can be derived in the following way:

Given the utility function which characterizes inequality aversion, in the first case (1) it holds that $\frac{\partial u_t^I(\pi_A,\Delta\pi)}{\partial\pi_B} = \frac{\partial u_t^I(\pi_A,\Delta\pi)}{\partial\Delta\pi_a}\cdot\frac{\partial\Delta\pi_a}{\partial\pi_B} > 0$.

(1a) If $\frac{\partial u_t^I(\pi_A,\overline{\Delta}\pi)}{\partial\pi_A} \geq -\frac{\partial u_t^I(\overline{\pi}_A,\Delta o)}{\partial\Delta\pi_a}\cdot\frac{\partial\Delta\pi_a}{\partial\pi_A}$ (this case reflects the assumption made by [1], p. 824 that the direct marginal utility of π_A always exceeds the indirect marginal loss caused by the greater payoff difference), then

$\frac{\partial u_t^I(\pi_A,\Delta\pi)}{\partial\pi_A} = \frac{\partial u_t^I(\pi_A,\overline{\Delta}\pi)}{\partial\pi_A} + \frac{\partial u_t^I(\overline{\pi}_A,\Delta\pi)}{\partial\Delta\pi_a}\cdot\frac{\partial\Delta\pi_a}{\partial\pi_A} \geq 0$ and the marginal rate of substitution (MRS) is non-positive, i.e. $\text{MRS} = -\frac{d\pi_B}{d\pi_A} = -\frac{\partial u_t^I(\pi_A,\Delta\pi)/\partial\pi_A}{\partial u_t^I(\pi_A,\Delta\pi)/\partial\pi_B} \leq 0$.

(1b) If $\frac{\partial u_t^I(\pi_A,\overline{\Delta}\pi)}{\partial\pi_A} < -\frac{\partial u_t^I(\overline{\pi}_A,\Delta\pi)}{\partial\Delta\pi_a}\cdot\frac{\partial\Delta\pi_a}{\partial\pi_A}$, then

$\frac{\partial u_t^I(\pi_A,\Delta\pi)}{\partial\pi_A} = \frac{\partial u_t^I(\pi_A,\overline{\Delta}\pi)}{\partial\pi_A} + \frac{\partial u_t^I(\overline{\pi}_A,\Delta\pi)}{\partial\Delta\pi_a}\cdot\frac{\partial\Delta\pi_a}{\partial\pi_A} < 0$ and the MRS is positive, i.e.

$\text{MRS} = -\frac{d\pi_B}{d\pi_A} = -\frac{\partial u_t^I(\pi_A,\Delta\pi)/\partial\pi_A}{\partial u_t^I(\pi_A,\Delta\pi)/\partial\pi_B} > 0$.

In the second case (2) it holds that $\frac{\partial u_t^I(\pi_A,\Delta\pi)}{\partial\pi_B} = \frac{\partial u_t^I(\pi_A,\Delta\pi)}{\partial\Delta\pi_d}\cdot\frac{\partial\Delta\pi_d}{\partial\pi_B} < 0$ and that $\frac{\partial u_t^I(\pi_A,\Delta\pi)}{\partial\pi_A} = \frac{\partial u_t^I(\pi_A,\overline{\Delta}\pi)}{\partial\pi_A} + \frac{\partial u_t^I(\overline{\pi}_A,\Delta\pi)}{\partial\Delta\pi_a}\cdot\frac{\partial\Delta\pi_a}{\partial\pi_A} \geq 0$. Consequently, the MRS is non-negative, i.e. $\text{MRS} = -\frac{d\pi_B}{d\pi_A} = -\frac{\partial u_t^I(\pi_A,\Delta\pi)/\partial\pi_A}{\partial u_t^I(\pi_A,\Delta\pi)/\partial\pi_B} \geq 0$.

Dictator games

Following our general notion of inequality aversion, in the give games, case (2) applies and the MRS is always larger than – p_A (i.e. the slope of the budget line). Consequently, an optimum choice will always be represented by a corner solution. Given that player A profits from a higher π_A and from a lower π_B, an I-consistent player A should keep everything to himself:

I-CONSISTENCY IN GIVE GAMES: An I-consistent player A in a give game will transfer no money to player B, i.e. $\gamma_{G1} = \gamma_{G2} = \gamma_{G3} = \gamma_{G4} = 0$.

Things are slightly more complicated if player A has a higher payoff than player B ($\pi_A \geq \pi_B$), as is the case in our take games. Changes in π_A will have two opposite effects. On the one hand, a rise in π_A increases utility. On the other hand, this also increases the inequality between π_A and π_B, which, in turn, decreases utility. Given that behavior is best described by (1a), then three different notions of I-consistency in the take games apply. First, if the MRS is greater than $-p_A^{T1}$, then player A should always take nothing. Second, if the MRS is less than $-p_A^{T4}$, then player A should always take everything. Third, if the MRS is less than $-p_A^{T1}$, but greater than $-p_A^{T4}$, then player A should not take more from player B as the relative price p_A of his own payoff increases. Note that these three notions require that preferences are homothetic. Given that behavior is best described by (1b), then I-consistency implies that player A always takes nothing since the MRS is greater than $-p_A^{T1}$.

I-CONSISTENCY IN TAKE GAMES: An I-consistent player A in a take game will not take more from B as the relative price p_A of his own payoff increases, i.e. $\tau_{T1} \geq \tau_{T2} \geq \tau_{T3} \geq \tau_{T4}$.

PD games

Given the previous remarks, for players A with inequality aversion preferences the definition of consistency in the prisoner's dilemma games is as follows:

I-CONSISTENCY IN PD GAMES: An I-consistent player A in both PD games who is following a *D* choice of player B (mini give games) should choose *d*. An I-consistent player A who is following a *C* choice of player B (mini take games) should choose always *d* or always *c* in both PD games or should choose *c* in PD I and *d* in PD II. All three notions of I-consistency in the mini take games result when (1b) holds. Otherwise, I-consistency implies that players A always choose *c* in both PD games.

In particular, the amount given to player B should be equal to zero (similar to S-consistency) and the amount taken from player B should never increase with p_A. For PD games this means that a player A who is following a *D* choice of player B should always choose *d* (similar to S-consistency) and a player A who is following a *C* choice of player B should always choose *d* or always choose *c* in both PD games or should choose *c* in PD I and *d* in PD II.

S1.3 Consistency according to altruistic preferences

Our second way of modeling other-regarding preferences is in the spirit of the concept introduced by [4]. According to this concept, one's own and the other's payoff are considered as 'normal goods'. Assuming that player A derives non-negative utility from her own payoff and from player B's payoff and ruling out the case of simultaneous indifference with regard to both, the utility function can be written as follows:

$$u_t^A(\pi_A, \pi_B) \quad \text{with} \quad \frac{\partial u_t^A}{\partial \pi_A} \geq 0, \frac{\partial u_t^A}{\partial \pi_B} \geq 0 \quad \text{and} \quad \exists i \in \{A, B\}, \text{such that } \frac{\partial u_t^A}{\partial \pi_i} > 0.$$

Using the additional assumption that neither π_A nor π_B are inferior, we conclude that 'demand' for π_A and π_B must each be falling in relation to its respective price. The implications for the definition of 'A-consistency' in our games can be characterized as follows:

Dictator games

Since π_A and π_B are normal goods, optimum demand for π_B should not decrease with the relative price p_A, i.e. $\partial \pi_B / \partial p_A \geq 0.$ Consequently, in the take games, the amount taken from player B, $\tau = 500 - \pi_B$, should not increase with p_A, i.e. $\partial(500 - \pi_B)/\partial p_A \leq 0.$ Given that in the four take games $p_A^{T1} < p_A^{T2} < p_A^{T3} < p_A^{T4}$, our definition of consistency in these games is:

A-CONSISTENCY IN TAKE GAMES: An A-consistent player A in a take game will not decrease π_B (will not take more from player B) as the relative price p_A of his own payoff increases, i.e.

$$\tau_{T1} \geq \tau_{T2} \geq \tau_{T3} \geq \tau_{T4}.$$

Accordingly, in the give games, the amount given to player B, $\gamma = \pi_B - 500$, should not fall with p_A, i.e. $\partial(\pi_B - 500)/\partial p_A \geq 0.$ Given that in the four give games $p_A^{G1} > p_A^{G2} > p_A^{G3} > p_A^{G4}$, our definition of consistency in the give games is:

A-CONSISTENCY IN GIVE GAMES: An A-consistent player A in a give game will not decrease π_B (will not give less to player B) as the relative price p_A of his own payoff increases, i.e.

$$\gamma_{G1} \geq \gamma_{G2} \geq \gamma_{G3} \geq \gamma_{G4}.$$

PD games

For players A with altruistic preferences, the definition of consistency in the prisoner's dilemma games is straightforward:

A-CONSISTENCY IN PD GAMES: An A-consistent player A who is following a *C* choice of player B (mini take games) should always choose *d* or always choose *c* in both PD games or should choose *c* in PD I and *d* in PD II.

An A-consistent player A who is following a *D* choice of player B (mini give games) should always choose *d* or always choose *c* in both PD games or should choose *d* in PD I and *c* in PD II.

In particular, the amount taken from player B should never increase with p_A (similar to I-consistency) and the amount given to player B should never decrease with p_A. For PD games this means that a player A who is following a *C* choice of player B should always choose *d* or always choose *c* in both PD games or should choose *c* in PD I and *d* in PD II (similar to I-consistency), and a player A who is following a *D* choice of player B should always choose *d* or always choose *c* in both PD games or should choose *d* in PD I and *c* in PD II. That is, for our games, I-consistency is a special case of A-consistency (and, accordingly, S-consistency is a special case of I- and A-consistency). All consistency definitions are summarized in Table A.

Table A: Definitions of consistency.

<table>
<tr><th></th><th>Give games
(γ_{Gi} denotes the amount given in give game *i*)</th><th>Take games
(τ_{Ti} denotes the amount taken in take game *i*)</th><th>PD I</th><th>PD II</th></tr>
<tr><td rowspan="2">S-consistency</td><td rowspan="2">$\gamma_{G1} = \gamma_{G2} = \gamma_{G3} = \gamma_{G4} = 0$</td><td rowspan="2">$\tau_{T1} = \tau_{T2} = \tau_{T3} = \tau_{T4} = 500$</td><td colspan="2">always *d*/*D*</td></tr>
<tr><td colspan="2">always *d*/*C*</td></tr>
<tr><td rowspan="2">I-consistency</td><td rowspan="2">$\gamma_{G1} = \gamma_{G2} = \gamma_{G3} = \gamma_{G4} = 0$</td><td rowspan="2">$\tau_{T1} \geq \tau_{T2} \geq \tau_{T3} \geq \tau_{T4}$</td><td colspan="2">always *d*/*D*</td></tr>
<tr><td colspan="2">always *d*/*C*,
always *c*/*C*,
c/*C* in PD I and *d*/*C* in PD II</td></tr>
<tr><td rowspan="2">A-consistency</td><td rowspan="2">$\gamma_{G1} \geq \gamma_{G2} \geq \gamma_{G3} \geq \gamma_{G4}$</td><td rowspan="2">$\tau_{T1} \geq \tau_{T2} \geq \tau_{T3} \geq \tau_{T4}$</td><td colspan="2">always *d*/*D*
always *c*/*D*,
d/*D* in PD I and *c*/*D* in PD II</td></tr>
<tr><td colspan="2">always *d*/*C*,
always *c*/*C*,
c/*C* in PD I and *d*/*C* in PD II</td></tr>
</table>

Consistency *across games* is defined as S-, A- or I-consistency across games.

S1.4 Results concerning consistency of individual behavior

Consistency within games

In order to investigate whether subjects behave consistently within any one of the three classes of games, we use the three concepts of consistency introduced in section 3. Note that, as already mentioned, these concepts are not independent measures. Since selfishness (S-consistency) is a special case of I-consistency and I-consistency is a special case of A-consistency in our games, the additional explanatory power of the latter concepts is limited to the cases where behavior is non-selfish or not I-consistent. Therefore, we should expect that most of the decisions are A-consistent and few are S-consistent. Fig A displays the results for all three waves:

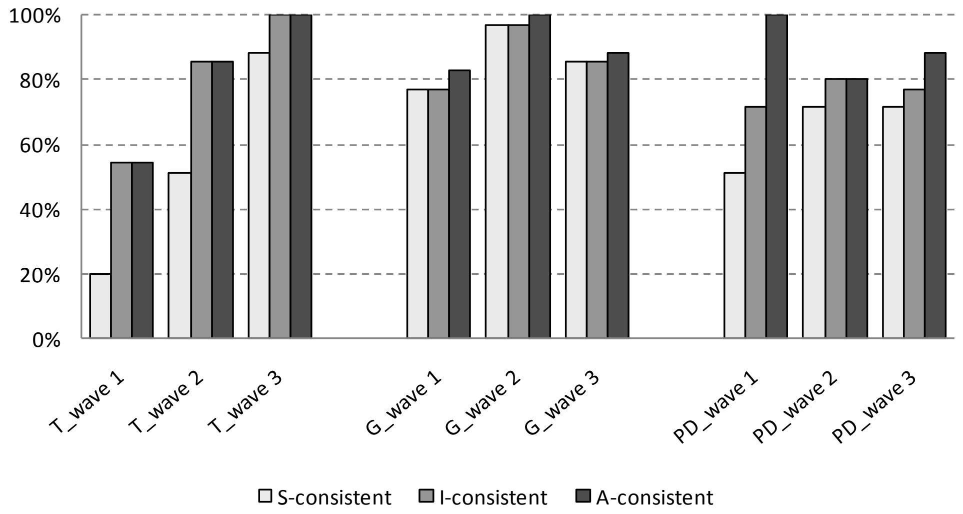

Fig A. Frequency of consistent behavior in all games in the three waves.

For the take games, the concepts of I- and A-consistency are identical and explain 54 percent of all individual moves in the first wave, whereas 20 percent of the subjects display consistently selfish behavior. That is, 34 percent of observed decisions in the first wave can additionally be interpreted as consistent when applying notions of consistency based on inequality aversion or altruism. In the second wave, we observe a highly significant increase in consistent behavior. Now, more than 85 percent of decisions are I- and A-consistent. The observation that the difference between the frequencies of selfish and other-regarding choices remains the same implies that the increase in consistency is mainly caused by the increase in the share of consistent selfishness. In the third wave, the share of consistently selfish behavior again increases significantly, this time to 89 percent. Only the decisions made by the 11 per-

cent of subjects who do not take all the money from players B can be accounted for by I- and A-consistency only. Remarkably, we do not observe any individual move in wave 3 which cannot be characterized as consistent in the sense of one of the three concepts.

In the give games, three quarters of the subjects already behave consistently selfishly in the first wave. This share significantly increases from wave 1 to wave 2, but does not significantly change from wave 2 to wave 3. S-consistency alone explains about 85 percent of the individual behavior observed in wave 3; there is no significant additional explanatory power offered by the other two concepts of consistency. Since those who decide to give something to player B choose, averaged over all of their decisions, only very small amounts, it seems to be adequate to characterize overall behavior in the give games as “consistently selfish”.

In the PD games, we find different patterns of behavior. All the subjects behave in an A-consistent manner in the first wave. The reason for the good performance of A-consistency is the fact that all players A follow one of the four A-consistent patterns: they always defect (18 of 35), or always cooperate (6 of 35), or always play tit-for-tat (7 of 35), or always play ‘inverted tit-for-tat’ (4 of 35), i.e. play *d*/*C* and *c*/*D*. Since I-consistent behavior is only in line with ‘always *d*’ and ‘tit-for-tat’, it could be observed in only 71 percent of all cases. 51 percent of the subjects behave strictly S-consistently and always decide to defect. In wave 2, the share of S-consistency significantly increases to 71 percent and remains at this level in wave 3. These shares are no longer significantly different from the shares of I- and A-consistency (for the latter this is only true in wave 2). The reason is that strategies that are not S-consistent (‘tit-for-tat’, ‘inverted tit-for-tat’, and ‘always *c*’) are hardly ever used any more. We summarize in observation S1-1:

Observation S1-1

The share of consistent moves within the three classes of games increases during the course of the experiment. This increase is almost always due to the fact that, over time, more and more subjects make consistently selfish decisions.

Consistency across games

The strongest test for the consistency concepts of individual behavior is the comparison of decisions made by any single subject in different games. Fig B summarizes our findings concerning the consistency over all three classes of games. We employ the definition of across-game consistency introduced in section S1.3 in all three cases.

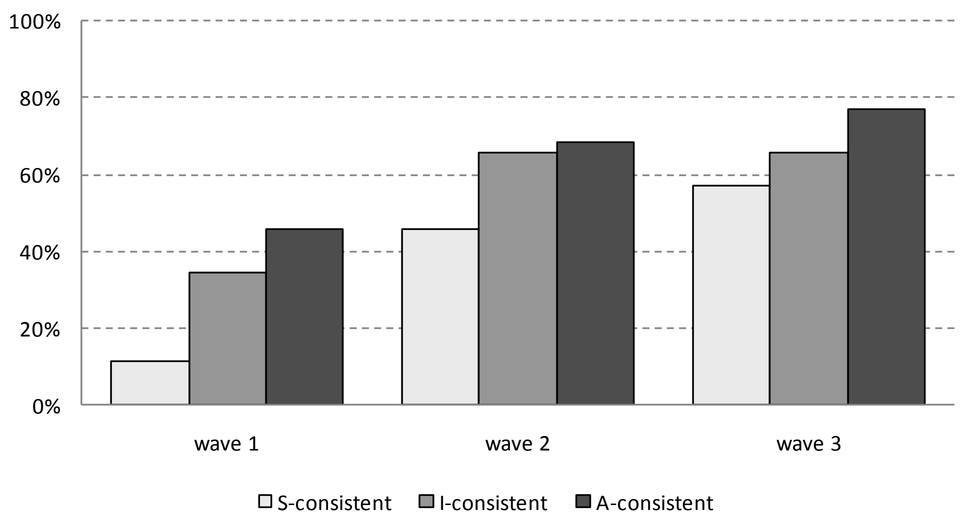

Fig B. Consistency across games.

In the first wave, only 11 percent of all the subjects exhibit consistently selfish behavior in all ten games. There are significantly more decisions that are I-consistent than S-consistent, though not significantly more that are A-consistent than I-consistent. A-consistency, the most general concept of consistency, accounts for the decisions made by 46 percent of the subjects. The latter observation implies that, in wave 1, more than 50 percent of all subjects behave *in*consistently over the three classes of games. In wave 2, overall inconsistency decreases to 31 percent, which is largely due to a significant increase in the frequency of S-consistent behavior from wave 1 to wave 2. Again, there is no significant difference between the number of I- and of A-consistent subjects. The shares of S-, I-, and A-consistent decisions do not significantly change from wave 2 to wave 3. We summarize in observation S1-2:

Observation S1-2

While in the first wave consistent selfishness is rarely observed across games, it dominates behavior in the second and the third waves. In the last wave, about three quarters of all decisions can be characterized as consistent across games.

Combining the observations on the behavioral dynamics with our three concepts of consistency reveals that there are only five out of 35 subjects who behave consistently across all games and do not change their behavior over the three waves. Four of these subjects behave strictly selfishly. Five subjects change their behavior from I-consistent to S-consistent and two subjects change their behavior from A-consistent to S-consistent over the three waves. That is, all *consistent* changes over the three waves are from less to more selfishness. In the last wave, 11 out of the 12 subjects revealing consistent decisions in all three waves can be

characterized as consistently selfish. A detailed discussion of individual (within-game) consistency and the dynamics of behavior can be found in S2 File.

Observation S1-3

One third of the subjects behave consistently across games and over all three waves; only one of them reveals consistent other-regarding behavior in the last wave.
