# Supplementary material for: The dynamics of behavior in modified dictator games
Source: PLoS One. 2017 Apr 27;12(4):e0176199. doi: 10.1371/journal.pone.0176199 (PMC5407812; doi:10.1371/journal.pone.0176199)
Supplement: S2 File — (PDF) [file pone.0176199.s002.pdf]

S2. Behavioral dynamics and consistency.

Fig A illustrates the results on individual across-game consistency and the dynamics of behavior.

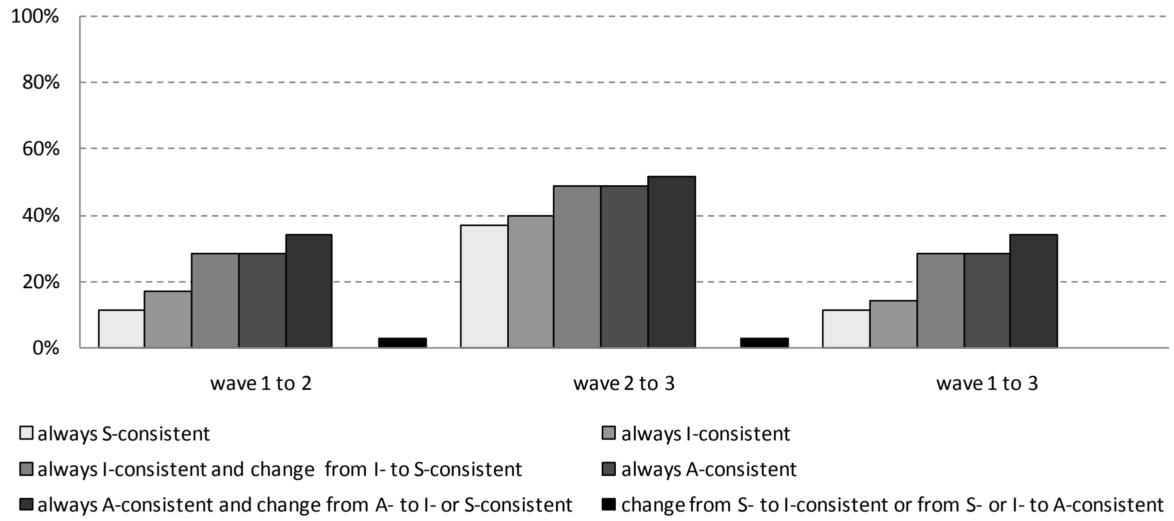

Fig A. Consistency across games and behavioral dynamics.

In the following, we analyze individual consistency and the dynamics of behavior, focusing on each class of games separately. In the take games, eight subjects behave consistently in all three waves and do not change their behavior; seven of them are consistently selfish. Ten subjects change from I-consistent to S-consistent behavior. That is, in wave 3, 17 out of the 18 subjects who behave consistently in all three waves reveal consistent selfishness. The results on take games are summarized in Fig B.

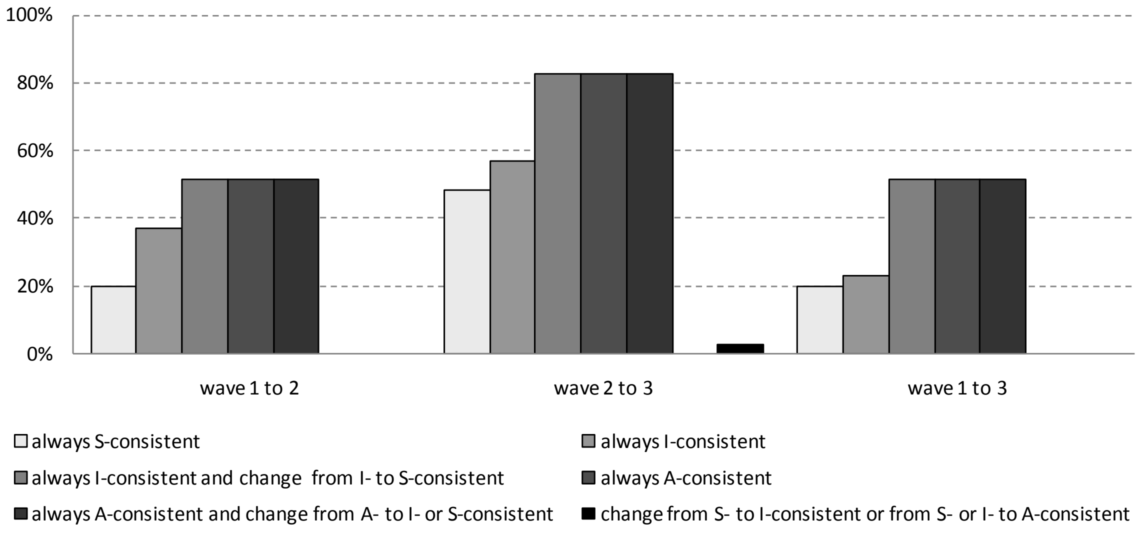

Fig B. Consistency and dynamics of behavior within take games.

In the give games, there is a great proportion of consistently and stable selfish behavior (23 subjects over all three waves). Only three subjects change their consistent behavior over the three waves, two of them switching from A-consistency (but not S- or I-consistency) to consistent selfishness, while one subject switches the other way around (see Fig C).

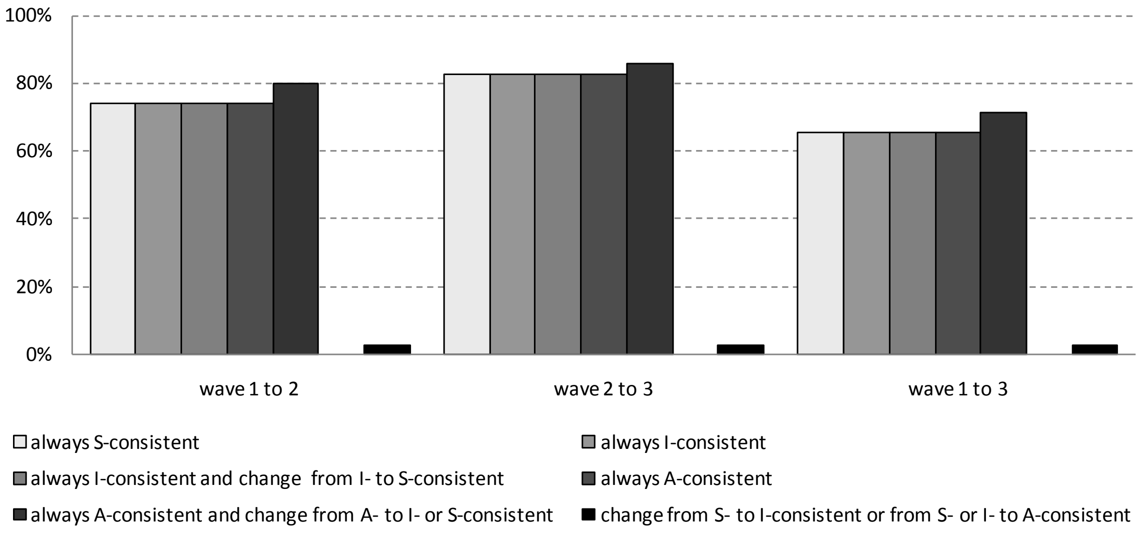

Fig C. Consistency and dynamics of behavior within give games

In the PD games, 16 subjects behave consistently and stable over the three waves, 15 of whom can be characterized as consistently selfish. Nine subjects change their consistent decisions, with four switching from I-consistency to S-consistency and five switching from A-

consistency to S-consistency. Again, in wave 3 the majority of subjects who behave consistently in all three waves are consistently selfish (see Fig D).

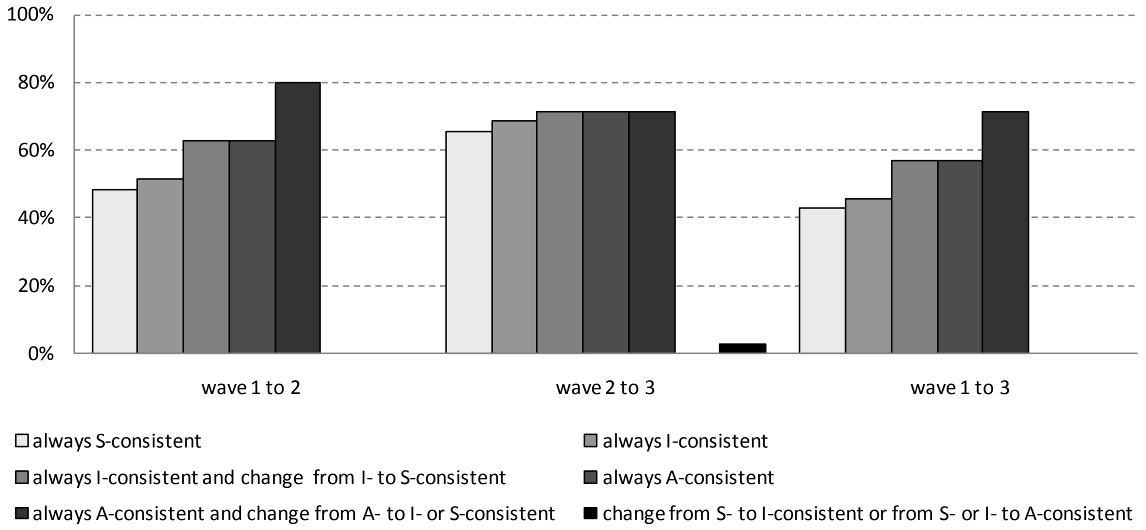

Fig D. Consistency and dynamics of behavior within PD games

Observation S2-1:

Restricting to within-game consistency, we observe that there are subjects switching from other-regarding to selfish behavior over time, but still making consistent decisions within each wave, particularly in the take and in the PD games. Nevertheless, stable S-consistency dominates in the PD games as well as in the give games.
