# Supplementary material for: The dynamics of behavior in modified dictator games
Source: PLoS One. 2017 Apr 27;12(4):e0176199. doi: 10.1371/journal.pone.0176199 (PMC5407812; doi:10.1371/journal.pone.0176199)
Supplement: S3 File — (PDF) [file pone.0176199.s003.pdf]

S3. Figures on the total payoff resulting from the decisions made by players A.

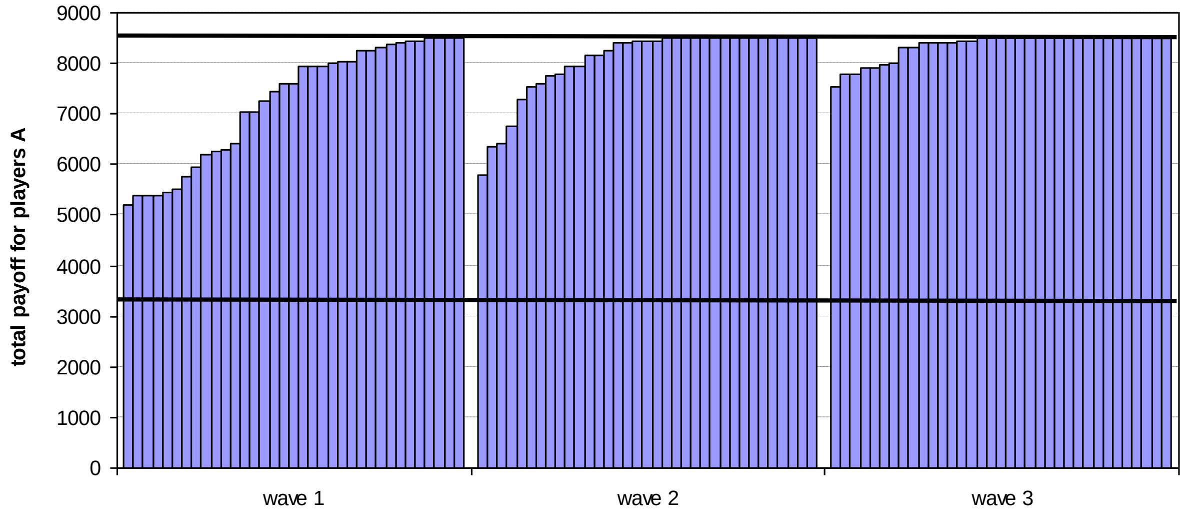

Fig A. **Total amount allocated by players A to themselves (includes all decisions by player A in the PD games; black lines indicate the maximum and minimum amounts).**

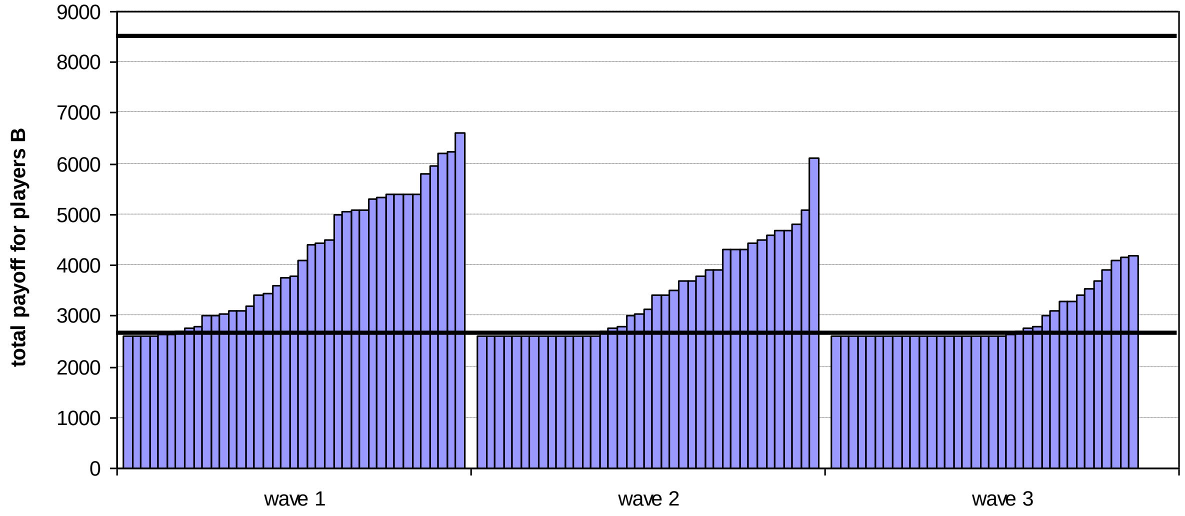

Fig B. **Total amount allocated by players A to player B (includes all decisions by player A in the PD games; black lines indicate the maximum and minimum amounts).**
