# Supplementary material for: The dynamics of behavior in modified dictator games
Source: PLoS One. 2017 Apr 27;12(4):e0176199. doi: 10.1371/journal.pone.0176199 (PMC5407812; doi:10.1371/journal.pone.0176199)
Supplement: S4 File — (PDF) [file pone.0176199.s004.pdf]

S4. Instructions.

(*for players A; similar instructions were handed out to players B*)

Note

You are participating in an investigation of individual decision behavior. If you have any questions which are not answered by these instructions, please let us know. We will come to you and answer your questions.

During this experiment you will earn money. It depends on your decisions during the experiment how much money this will be. At the end of the experiment, the money will be paid to you in room C-214 when you display your ID card there.

Decision

During the experiment, you will have to make decisions at the computer. Before every decision, you will be given detailed instructions on the computer screen.

There is one other participant involved in each of your decision situations. This other participant will be newly allocated to you in each of your decision situations. We have made sure that you will interact with one and the same participant only once. No participant learns the identity of his allocated partners either during or after the experiment. Your decisions remain **anonymous**.

Please keep in mind that your decision situations are independent of one another, which means that none of your decisions has an influence on the other decisions.

[*Wave 3 only:* In the appendix, you will find a list of the decision situations you will be confronted with during this experiment.]

Payoff

At the end of the experiment, we will compute your payoff in the laboratory. The exchange rate of laboratory cents to eurocents is 150 laboratory cents = 100 eurocents. The payoff to each of the participants will be put into an envelope, the envelope will be closed and labeled with the respective ID number. The envelope will then be brought to room C-214 where a member of the staff who was not involved in the computation of the payoffs and who is sitting behind a blind will hand out your payoff when you display your ID card. This procedure makes sure that your decisions remain anonymous vis-a-vis the other participants and vis-a-vis the experimenter.

Please do not communicate with the other participants during the experiment. Moreover, we would ask you not to talk about the experiment to others in order to avoid influencing the behavior of potential future participants.

We thank you for your participation.

Decision situation 1:

Here you can determine the payoff to yourself and the payoff to your partner. Please choose one of the following combinations of payoffs:

choice:	○	You:	500	Partner:	500
	○	You:	450	Partner:	550
	○	You:	400	Partner:	600
	○	You:	350	Partner:	650
	○	You:	300	Partner:	700
	○	You:	250	Partner:	750
	○	You:	200	Partner:	800
	○	You:	150	Partner:	850
	○	You:	100	Partner:	900
	○	You:	50	Partner:	950
	○	You:	0	Partner:	1000

Decision situation 2:

Here you can determine the payoff to yourself and the payoff to your partner. Please choose one of the following combinations of payoffs:

choice:	○	You:	500	Partner:	500
	○	You:	450	Partner:	600
	○	You:	400	Partner:	700
	○	You:	350	Partner:	800
	○	You:	300	Partner:	900
	○	You:	250	Partner:	1000
	○	You:	200	Partner:	1100
	○	You:	150	Partner:	1200
	○	You:	100	Partner:	1300
	○	You:	50	Partner:	1400
	○	You:	0	Partner:	1500

Decision situation 3:

Please choose one of the “strategies” A or B for every possible choice of your partner. Your payoff depends on what you choose and what your partner chooses.

The following table displays your payoffs. If your partner chooses “A” and you choose “A if partner chooses A”, your payoff is 500 (Your payoff is given by the second entry of the respective cell in the table given below.) If your partner chooses “A” and you choose “B if partner chooses A”, your payoff is 900 and the payoff of your partner is 100. If your partner chooses “B” and you choose “”A if partner chooses B”, your payoff is 100 and the payoff of your partner is 900. In the case that your partner chooses “B” and you choose “B if partner chooses B”, your payoff is 200 and the payoff of your partner is also 200.

	you choose A	you choose B
Your partner chooses A	500 , 500	100 , 900
Your partner chooses B	900 , 100	200 , 200

Your choice, if partner chooses “A” ○ A
○ B

Your choice, if partner chooses “B” ○ A
○ B

Decision situation 4:

Here you can determine the payoff to yourself and the payoff to your partner. Please choose one of the following combinations of payoffs:

choice:	○	You: 500	Partner: 500
	○	You: 450	Partner: 525
	○	You: 400	Partner: 550
	○	You: 350	Partner: 575
	○	You: 300	Partner: 600
	○	You: 250	Partner: 625
	○	You: 200	Partner: 650
	○	You: 150	Partner: 675
	○	You: 100	Partner: 700
	○	You: 50	Partner: 725
	○	You: 0	Partner: 750

Decision situation 5:

Here you can determine the payoff to yourself and the payoff to your partner. Please choose one of the following combinations of payoffs:

choice:	○	You:	500	Partner:	500
	○	You:	450	Partner:	575
	○	You:	400	Partner:	850
	○	You:	350	Partner:	925
	○	You:	300	Partner:	1000
	○	You:	250	Partner:	1025
	○	You:	200	Partner:	1050
	○	You:	150	Partner:	1125
	○	You:	100	Partner:	1200
	○	You:	50	Partner:	1225
	○	You:	0	Partner:	1250
