# Supplementary material for: The dynamics of behavior in modified dictator games
Source: PLoS One. 2017 Apr 27;12(4):e0176199. doi: 10.1371/journal.pone.0176199 (PMC5407812; doi:10.1371/journal.pone.0176199)
Supplement: S5 File — (PDF) [file pone.0176199.s005.pdf]

S5. Data.

Table A. Individual τ in take games

wave	1				2				3			
**game**	**T1**	**T2**	**T3**	**T4**	**T1**	**T2**	**T3**	**T4**	**T1**	**T2**	**T3**	**T4**
**subject**												
**1**	350	450	350	450	500	500	500	500	500	500	500	500
**2**	500	500	500	500	500	500	500	500	500	500	500	500
**3**	500	500	50	0	200	0	500	0	500	500	500	500
**4**	0	0	0	0	500	500	400	500				
**5**	0	0	0	400	500	500	500	500	500	500	500	500
**6**	0	400	50	0	500	500	500	0	500	500	500	500
**7**	0	0	50	0	0	500	0	0	500	500	500	500
**8**	450	400	350	200	500	500	500	500	500	500	500	500
**9**	0	200	50	500	500	500	500	0	500	500	500	500
**10**	200	250	200	200								
**11**	500	500	500	500	500	500	500	500	500	500	500	500
**12**	450	500	500	500	500	500	500	500	500	500	500	500
**13**	0	0	500	0	0	0	0	0				
**14**	450	450	450	450	450	450	450	450	500	500	450	450
**15**	100	150	100	50	400	400	400	400	500	500	500	500
**16**	100	50	100	150	200	500	200	500	500	500	500	500
**17**	500	500	500	500	500	500	500	500	500	500	500	500
**18**	0	200	0	0	500	500	500	500	500	500	500	500
**19**	500	500	450	450	500	500	500	500	500	500	500	500
**20**	0	0	0	0	0	0	0	0	500	500	500	500

**21**	250	200	400	500	500	500	450	450	500	500	500	500
**22**	500	500	500	500	500	500	500	500	500	500	500	500
**23**	500	450	500	500	500	500	250	300	500	500	500	500
**24**	500	450	400	350	500	500	500	400	500	500	500	500
**25**	500	500	500	500	500	500	500	500	500	500	500	500
**26**	0	0	0	0	500	500	450	400	500	500	500	500
**27**	500	500	250	400	500	500	500	500	500	500	500	500
**28**	500	500	200	0	500	500	500	500	500	500	500	500
**29**	500	300	300	500	500	500	500	500	500	500	500	500
**30**	500	500	500	500	500	500	500	500	500	500	500	500
**31**	200	250	0	0	300	250	150	0	350	350	300	200
**32**	500	500	500	0	500	500	500	400	500	500	500	500
**33**	0	100	50	50	0	150	100	50	500	500	500	500
**34**	0	0	0	0	500	500	450	200	500	500	500	500
**35**	200	300	300	0	450	450	400	350	500	450	450	400
**36**	500	500	500	500	500	500	500	500	500	500	500	500
**37**	500	500	400	450	500	500	500	350	500	500	500	400
**38**	500	500	500	0	500	500	500	500	500	500	500	500
**39**	400	400	200	0	500	500	500	500	500	500	500	500
**40**	500	300	400	0	500	500	500	500	500	500	450	450

Table B. Individual γ in give games

wave	1				2				3			
**game**	**G1**	**G2**	**G3**	**G4**	**G1**	**G2**	**G3**	**G4**	**G1**	**G2**	**G3**	**G4**
**subject**												
**1**	0	0	0	0	0	0	0	0	0	0	0	0
**2**	0	75	0	0	0	0	0	0	0	0	0	0
**3**	0	75	0	50	0	0	0	0	0	0	0	0
**4**	0	0	0	0	0	0	0	0				
**5**	0	0	0	0	0	0	0	0	0	0	0	0
**6**	200	0	0	0	0	0	0	0	0	0	0	0
**7**	0	0	0	0	0	0	0	0	0	0	0	0
**8**	0	0	0	0	0	0	0	0	0	0	0	0
**9**	0	0	0	0	0	0	0	0	0	0	0	0
**10**	0	0	0	0								
**11**	0	0	500	0	0	0	0	0	0	0	0	0
**12**	0	0	0	0	0	0	0	0	0	0	0	0
**13**	1000	0	0	250	0	0	0	0				
**14**	0	0	0	0	0	0	0	0	0	0	0	0
**15**	0	0	0	0	0	0	0	0	0	0	0	0
**16**	0	0	0	0	0	0	0	0	0	0	0	0
**17**	0	0	0	0	0	0	0	0	0	0	0	0
**18**	0	0	0	0	0	0	0	0	0	0	0	0
**19**	0	0	0	0	0	0	0	0	0	0	0	0
**20**	0	0	0	0	0	0	0	0	0	0	0	0
**21**	0	300	0	0	0	0	0	0	0	750	450	0
**22**	0	0	0	0	0	0	0	0	0	0	0	0

**23**	0	0	400	0	0	0	0	0	0	0	0	0
**24**	0	0	0	0	0	0	0	0	0	0	0	0
**25**	0	0	0	0	0	0	0	0	0	0	0	0
**26**	0	0	0	0	0	0	0	0	0	0	0	0
**27**	0	0	0	0	0	0	0	0	0	0	0	25
**28**	0	0	0	0	0	0	0	0	0	0	0	0
**29**	0	0	0	0	0	0	0	0				
**30**	0	0	0	0	0	0	0	0	0	0	0	0
**31**	400	150	0	0	0	0	0	0				
**32**	0	0	0	0	0	0	0	0	0	0	0	0
**33**	100	75	0	0	0	0	0	0	0	0	0	0
**34**	0	0	0	0	200	75	0	0	0	0	0	0
**35**	0	75	0	50	0	0	0	0	100	150	0	0
**36**	0	0	0	0	0	0	0	0	0	0	0	0
**37**	0	0	0	0	0	0	0	0	0	75	0	0
**38**	0	0	0	0	0	0	0	0	0	0	0	0
**39**	0	0	0	0	0	0	0	0	0	0	0	0
**40**	0	0	0	0	0	0	0	0	100	75	0	0

Table C. Individual action choices in PD games

wave	1				2				3			
game	PD I		PD II		PD I		PD II		PD I		PD II	
	if *C*	if *D*	if *C*	if *D*	if *C*	if *D*	if *C*	if *D*	if *C*	if *D*	if *C*	if *D*
subject												
1	*d*	*d*	*d*	*d*	*d*	*d*	*d*	*d*	*d*	*d*	*c*	*c*
2	*d*	*d*	*d*	*d*	*d*	*d*	*d*	*d*	*d*	*d*	*d*	*d*
3	*c*	*d*	*c*	*d*	*d*	*d*	*c*	*c*	*d*	*c*	*c*	*c*
4	*d*	*c*	*d*	*c*	*d*	*d*	*d*	*d*				
5	*c*	*c*	*c*	*c*	*d*	*d*	*d*	*d*	*d*	*d*	*d*	*d*
6	*c*	*c*	*c*	*c*	*c*	*c*	*c*	*d*	*c*	*d*	*c*	*c*
7	*c*	*d*	*c*	*d*	*d*	*d*	*d*	*d*	*d*	*d*	*d*	*d*
8	*d*	*d*	*d*	*d*	*d*	*d*	*d*	*d*	*d*	*d*	*d*	*d*
9	*d*	*c*	*d*	*c*	*d*	*c*	*d*	*d*	*d*	*d*	*d*	*c*
10	*d*	*d*	*d*	*c*								
11	*d*	*d*	*d*	*d*	*d*	*d*	*d*	*d*	*d*	*d*	*d*	*d*
12	*d*	*d*	*d*	*d*	*d*	*d*	*d*	*d*	*d*	*d*	*d*	*d*
13	*d*	*d*	*d*	*d*	*d*	*d*	*d*	*d*				
14	*d*	*d*	*d*	*d*	*d*	*d*	*d*	*d*	*d*	*d*	*d*	*d*
15	*c*	*d*	*c*	*d*	*c*	*c*	*c*	*d*	*d*	*d*	*d*	*c*
16	*d*	*c*	*d*	*c*	*d*	*d*	*d*	*d*	*d*	*d*	*d*	*d*
17	*d*	*d*	*d*	*d*	*d*	*d*	*d*	*d*	*d*	*d*	*d*	*d*
18	*d*	*d*	*d*	*d*	*d*	*d*	*d*	*d*	*d*	*d*	*d*	*d*
19	*d*	*d*	*d*	*d*	*d*	*d*	*d*	*d*	*d*	*d*	*d*	*d*
20	*c*	*c*	*c*	*c*	*c*	*d*	*d*	*d*	*d*	*d*	*d*	*d*
21	*d*	*d*	*d*	*d*	*d*	*d*	*d*	*d*	*d*	*d*	*d*	*d*

**22**	*d*	*d*	*d*	*d*	*d*	*d*	*d*	*d*	*d*	*d*	*d*	*d*
**23**	*d*	*d*	*d*	*d*	*d*	*d*	*d*	*d*	*c*	*c*	*c*	*d*
**24**	*d*	*c*	*d*	*c*	*d*	*d*	*d*	*d*	*d*	*d*	*d*	*d*
**25**	*d*	*c*	*d*	*c*	*d*	*c*	*d*	*d*	*d*	*c*	*d*	*d*
**26**	*c*	*d*	*c*	*d*	*d*	*d*	*d*	*d*	*d*	*d*	*d*	*d*
**27**	*c*	*d*	*c*	*d*	*d*	*d*	*d*	*d*	*d*	*d*	*d*	*d*
**28**	*d*	*d*	*d*	*d*	*c*	*c*	*c*	*d*	*d*	*d*	*d*	*d*
**29**	*d*	*d*	*d*	*d*	*d*	*d*	*d*	*d*	*d*	*d*		
**30**	*d*	*d*	*d*	*d*	*d*	*d*	*d*	*d*	*d*	*d*	*d*	*d*
**31**	*d*	*d*	*d*	*d*	*c*	*c*	*d*	*c*	*c*	*c*		
**32**	*c*	*c*	*c*	*c*	*c*	*c*	*c*	*d*	*c*	*d*	*c*	*d*
**33**	*c*	*d*	*c*	*d*	*d*	*d*	*d*	*d*	*d*	*d*	*d*	*d*
**34**	*c*	*d*	*c*	*d*	*c*	*d*	*d*	*d*	*c*	*d*	*d*	*d*
**35**	*c*	*c*	*c*	*c*	*c*	*d*	*d*	*d*	*d*	*c*	*d*	*c*
**36**	*d*	*d*	*d*	*d*	*d*	*d*	*d*	*d*	*d*	*d*	*d*	*d*
**37**	*d*	*d*	*d*	*d*	*d*	*d*	*d*	*d*	*d*	*d*	*d*	*d*
**38**	*d*	*d*	*d*	*d*	*d*	*d*	*d*	*d*	*d*	*d*	*d*	*d*
**39**	*d*	*d*	*d*	*d*	*d*	*d*	*d*	*d*	*d*	*d*	*d*	*d*
**40**	*c*	*c*	*c*	*c*	*d*	*d*	*d*	*d*	*d*	*d*	*d*	*d*
