# Supplementary material for: The dynamics of behavior in modified dictator games
Source: PLoS One. 2017 Apr 27;12(4):e0176199. doi: 10.1371/journal.pone.0176199 (PMC5407812; doi:10.1371/journal.pone.0176199)
Supplement: S6 File — (PDF) [file pone.0176199.s006.pdf]

S6. Regression results.

Table A. Ordered probit model on the amount taken from player B.

	Ordered Probit
Wave 2	0.837***<br>(0.176)
Wave 3	2.006***<br>(0.247)
T2	0.0718<br>(0.0939)
T3	-0.252**<br>(0.100)
T4	-0.552***<br>(0.149)
Observations	420
Cluster per subjects	yes
Prob > Chi2	0.0000
Pseudo R^2	0.1310

Notes: Ordered Probit regression. Standard errors in parentheses, clustered at subjects level.
***Significant at the 1 percent level.
**Significant at the 5 percent level.
* Significant at the 10 percent level

Table B. Ordered logit model on the amount taken from player B.

	Ordered Logit
Wave 2	1.439***<br>(0.296)
Wave 3	3.461***<br>(0.502)
T2	0.0832<br>(0.157)
T3	-0.501***<br>(0.184)
T4	-0.986***<br>(0.276)
Observations	420
Cluster per subjects	yes
Prob > Chi2	0.0000
Pseudo R^2	0.1289

Notes: Ordered logit regression. Standard errors in parentheses, clustered at subjects level.
***Significant at the 1 percent level.
**Significant at the 5 percent level.
* Significant at the 10 percent level

Table C. Hurdle model on the amount given to player B.

	Logit Participate	Truncated linear regression Tau
Wave 2	-0.813*	-6.257
	(0.454)	(164.1)
Wave 3	-0.177	165.7
	(0.252)	(172.6)
T2	0.359*	126.4
	(0.195)	(324.1)
T3	-0.270	650.5**
	(0.342)	(290.5)
T4	-0.270	-1202.2
	(0.294)	(943.1)
Constant	-1.414***	-283.9
	(0.232)	(423.8)
Observations	420	21(!)
Cluster per subjects	yes	yes

Notes: Hurdle model on amount given to Player B. Standard errors in parentheses, clustered at subjects level. Wald test has Chi2(5)=32.17 (p-value<0.001)
***Significant at the 1 percent level.
**Significant at the 5 percent level.
* Significant at the 10 percent level

Table D. Ordered probit model on the amount given to player B.

	Ordered Probit
Wave 2	-0.792*
	(0.438)
Wave 3	-0.142
	(0.245)
G2	0.326
	(0.216)
G3	-0.180
	(0.341)
G4	-0.352
	(0.278)
Observations	420
Cluster per subjects	yes
Prob > Chi2	0.0000
Pseudo R^2	0.0527

Notes: Ordered probit regression on amount given to player B. Standard errors in parentheses, clustered at subjects level.
***Significant at the 1 percent level.
**Significant at the 5 percent level.
* Significant at the 10 percent level

Table E. Ordered logit model on the amount given to player B.

	Ordered Logit
Wave 2	-1.784
	(1.111)
Wave 3	-0.329
	(0.517)
G2	0.734*
	(0.431)
G3	-0.516
	(0.800)
G4	-0.584
	(0.658)
Observations	420
Cluster per subjects	yes
Prob > Chi2	0.0000
Pseudo R^2	0.0524

Notes: Ordered logit regression on amount given to player B. Standard errors in parentheses, clustered at subjects level.
***Significant at the 1 percent level.
**Significant at the 5 percent level.
* Significant at the 10 percent level

Table F. Logit model on decisions made in the PD games.

	Logit regression
Wave 2	0.663***
	(0.200)
Wave 3	0.655***
	(0.214)
PD II	0.0772
	(0.0969)
B chooses D	0.177
	(0.168)
Constant	0.319
	(0.213)
Observations	240
Cluster per subjects	yes
Prob > Chi2	0.0065
Pseudo R^2	0.0535

Notes: Logit regression on choosing option *c* or *d* with *c*=0 and *d*=1. Standard errors in parentheses, clustered at subjects level.
***Significant at the 1 percent level.
**Significant at the 5 percent level.
* Significant at the 10 percent level

Table G. Probit model on decisions made in the PD games.

	Probit regression
Wave 2	1.142***<br>(0.354)
Wave 3	1.142***<br>(0.379)
PD II	0.126<br>(0.162)
B chooses D	0.315<br>(0.299)
Constant	0.499<br>(0.350)
Observations	240
Cluster per subjects	yes
Prob > Chi2	0.0071
Pseudo R^2	0.0535

Notes: Probit regression on choosing option *c* or *d* with *c*=0 and *d*=1. Standard errors in parentheses, clustered at subjects level.
***Significant at the 1 percent level.
**Significant at the 5 percent level.
* Significant at the 10 percent level
